# Portfolio analysis of global tobacco control research funding at the National Cancer Institute, 2000–2019

**DOI:** 10.18332/tpc/184041

**Published:** 2024-03-04

**Authors:** Marie D. Ricciardone, Laura Baker, Jenny Twesten, Mark Parascandola

**Affiliations:** 1 Center for Global Health, National Cancer Institute, Bethesda, MD 20892, USA; 2 Strategix Management, LLC, Washington, DC 20036, USA; 3 The Bizzell Group, LLC, New Carrollton, MD 20785, USA

**Keywords:** tobacco control, global health, LMICs, portfolio analysis, research funding, research capacity building

## Abstract

**INTRODUCTION:**

Research in low- and middle-income countries (LMICs), where the majority of global tobacco users reside, is critical to addressing the global tobacco epidemic. This analysis describes the global tobacco control research portfolio funded by the National Cancer Institute from fiscal years 2000 to 2019.

**METHODS:**

We used the National Institutes of Health Query, View, Report database to identify extramural grants relevant to global tobacco control research. Abstracts were analyzed to describe grant characteristics, including topic areas, tobacco products, countries, and regions of focus. Bibliometric and co-authorship network analyses were performed for publications associated with relevant grants.

**RESULTS:**

Of the 93 relevant grants with foreign (non-US) involvement, the majority (83.9%) supported research in upper and lower middle-income countries. The majority of grants (86.0%) focused on cigarettes, with a small subset of grants addressing smokeless tobacco, waterpipe use, or other non-cigarette products. Most grants focused on at least one of the six tobacco control policy measures in the World Health Organization MPOWER package; almost half (48.4%) focused on monitoring tobacco use and around one-third (32.3%) focused on offering tobacco cessation treatment, while other MPOWER measures received less attention in the research portfolio. While most of these grants, and the funding initiatives that supported them, emphasized research in low- and middle-income countries (LMICs), only 3 of 93 grants were awarded directly to LMIC-based institutions.

**CONCLUSIONS:**

There is a critical need for research to develop and test strategies to adapt, implement, and scale up evidence-based interventions across diverse LMIC settings. This study identified gaps in research activity that should be addressed to strengthen global tobacco control research capacity.

## INTRODUCTION

While there has been progress in reducing tobacco use through the implementation of evidence-based programs and policies over the past half-century, tobacco use remains a global challenge. In 2019, the prevalence of smoking tobacco among individuals aged ≥15 years was 32.7% among males and 6.6% among females. At the same time, 7.69 million deaths were attributable to smoking tobacco; 80% of these deaths were among males, and 77.5% of these deaths occurred in low-income and middle-income countries (LMICs)^1^. The future burden of tobacco-related morbidity and mortality will be borne primarily by LMICs^2^, and two World Health Organization (WHO) regions, the African Region and the Eastern Mediterranean Region, are projected to see increases in the overall number of tobacco smokers^3^.

As of 2023, the vast majority of countries (182) are parties to the WHO Framework Convention on Tobacco Control (FCTC), though the United States is not^4^. In 2008, the WHO introduced the MPOWER measures, a set of six evidence-based tobacco control policy measures to help countries implement the WHO FCTC^5^. The 2023 WHO Report on the Global Tobacco Epidemic^6^ states that 65% of the world’s population and 61% of those living in LMICs are covered by at least one MPOWER measure (not including *Monitor*) at the highest level of achievement, a substantial increase over the previous decade. However, the adoption and implementation of some MPOWER measures remain incomplete. For example, although many countries offer some form of tobacco cessation support in some settings, the accessibility and coverage of such services are often limited^7,8^.

Research plays a critical role in addressing the global tobacco epidemic. The advances seen in high-income countries (HICs) in reducing tobacco use and related mortality over previous decades were largely a result of the implementation of evidence-based policies, programs, and interventions^9^. However, although a substantial body of evidence exists to support a range of cessation interventions for the general public^10,11^, most of this evidence comes from HICs and is only partly applicable to the evolving social, economic, and cultural climate of many LMICs, which have diverse health systems, tobacco use behaviors, patterns of dependence, and tobacco product markets^2^. A 2019 systematic review found few studies of the effectiveness of behavioral and pharmacologic treatments for tobacco dependence conducted in LMICs^12^. To be effectively implemented, tobacco control interventions and policies may need to be adapted to LMIC settings in terms of their unique social, cultural, political, economic, and regulatory contexts; tobacco product availability and use patterns; and tobacco industry influence, among other factors^13,14^. Furthermore, research in LMICs can provide valuable insights to inform tobacco control efforts in HICs, such as through addressing health disparities, developing and testing low-cost cessation interventions, and informing the implementation of graphic warning labels^14^.

The U.S. National Institutes of Health (NIH) is a major funder of international tobacco control research (along with other funding agencies, including the Bill and Melinda Gates Foundation, the American Cancer Society, Cancer Research UK, Bloomberg Philanthropies, and Canada’s International Development Research Centre)^15^. In 2002, NIH’s Fogarty International Center (FIC), in partnership with the National Cancer Institute (NCI) and National Institute on Drug Abuse (NIDA), established the International Tobacco and Health Research and Capacity Building (TOBAC) program to support collaborative research, and research and capacity-building projects that address the burden of tobacco use in LMICs^16^. The TOBAC program made 41 awards over four funding cycles in 2002, 2007, 2012, and 2017^16,17^. NCI also supports a broad portfolio of tobacco control research conducted outside the US through funding announcements related to tobacco regulatory science, tobacco use, and HIV in LMICs, polysubstance abuse and addiction, and other topics^18^. Given that tobacco use remains a leading cause of preventable cancer deaths around the world^19^, addressing tobacco use internationally is a priority for cancer research and control communities.

Understanding the scope and reach of NCI funding for global tobacco control research can help identify research gaps and opportunities, and can inform future funding initiatives to address tobacco use in LMICs. The current analysis describes NCI’s extramural global tobacco control portfolio from fiscal years 2000 to 2019. We also included all grants supported through the TOBAC program during this period, given the global focus and NCI involvement. We sought to characterize which countries and regions were most represented in the portfolio, which research topic areas were represented, what proportion of grants went to US versus non-US institutions, and the characteristics of collaborations and publications between US and non-US scientists.

## METHODS

### Search strategy

We used the NIH Query, View, Report (QVR) database to identify extramural grants funded by NCI or FIC as the primary or secondary funders between fiscal year (FY) 2000 and FY 2019. QVR is an internal database used by NIH program officials and not accessible to the public, though some data about individual grants can be accessed through the public NIH Reporter website (https://reporter.nih.gov). Grant titles and abstracts were searched for the terms: ‘smoking’ OR ‘cigarette’ OR ‘tobacco’ OR ‘cigar’ OR ‘smokeless’ OR ‘tobacco control’. The search criteria included all grants with foreign (non-US) country involvement, including direct foreign awards and awards to US institutions with foreign components.

### Inclusion criteria

Grants were included in the analysis if they were funded by NCI or FIC as the primary or secondary funder or were awarded through the TOBAC program; were active during the time period; included an international component (e.g. at least one foreign performance site in the Foreign Award and Component Tracking System, an abstract that denoted relevance to one or more foreign countries); and had one or more specific aims related to tobacco control. Grants could be co-funded by other NIH Institutes or other Government agencies (e.g. NIDA, Food and Drug Administration) in addition to NCI and FIC. Grant titles and abstracts were reviewed by two coders. When it was unclear if there was a focus on tobacco control, a third coder determined whether the grant was relevant.

### Data extraction

Administrative data about relevant grants were extracted from the QVR, including funding institutions or agencies, the Request for Applications (RFA) number and title, grant number and title, mechanism, start and end dates, principal investigator (PI), PI institution, performance sites and foreign sites named within the grant record, and publication records.

The applicable WHO regions and FY 2020 World Bank country income categories were assigned based on the grant’s research focus (as described in the grant abstract) and on the countries of the foreign sites named in each grant record; in other words, the grant was assigned a country location based on where the research was taking place, rather than where the primary grantee institution was located. For consistency across the analysis, we applied 2020 World Bank income classifications to all awards, although income classifications have changed for some countries over time.

Grants were also coded manually for other characteristics, including the use of research methods and areas of research focus. Two coders conducted qualitative coding based on the grant abstract for the categories below. We double-coded to ensure agreement across variables and resolved discrepancies through group discussions. Where the grant abstract did not yield enough information, the grant application was examined as available to the authors through NCI systems.

### Coding categories

- Stated emphasis on special or vulnerable populations who are particularly at-risk or understudied, such as populations with communicable diseases; maternal and child health populations (e.g. women of reproductive age, women who are pregnant or breastfeeding, or newborns; individuals or groups of low socioeconomic status (SES); youths and young adults; and health professionals, including physicians, nurses, and students in a health-related field.
- Tobacco product(s) studied (e.g. cigarettes, hookah/waterpipe, smokeless tobacco, e-cigarettes/electronic nicotine delivery systems [ENDS], other tobacco products [e.g. cigars, bidi, IQOS], or secondhand smoke).
- Research focus based on WHO’s MPOWER framework (*Monitor* tobacco use and prevention policies; *Protect* people from tobacco smoke; *Offer* help to quit tobacco use; *Warn* about the dangers of tobacco; *Enforce* bans on tobacco advertising, promotion, and sponsorship; and *Raise* taxes on tobacco)^20^, and supplemented by other WHO FCTC relevant areas^21^, including tobacco trade and farming (Articles 17 and 18), tobacco product constituents (Articles 9 and 10), the health effects of tobacco, tobacco industry interference (Article 5.3), or other.
- A stated research training component is defined as classroom-based programs, seminars, workshops, or a mentorship.
- Study designs used, including survey (cross-sectional or longitudinal), secondary data analysis, epidemiologic study (case-control or cohort), intervention, laboratory study (environmental/air analysis or tobacco product constituents), qualitative research, modeling, analysis of tobacco industry documents, another type of design, or unspecified design.

### Analysis

Extracted data were summarized using frequencies. To assess the bibliometric data, the PubMed reference numbers (PMIDs) associated with each grant were input to iSearch^22^ and iCite (NIH tools for portfolio and bibliometric analysis) to calculate the Relative Citation Ratio (RCR), benchmarked to 1.0 for a typical (median) NIH article in the corresponding year of publication^23^. PMIDs were used rather than PMCID to provide consistency over the 20-year time period. The bibliometric analysis included all PMIDs associated with each grant. The co-authorship network analysis of the Eastern Mediterranean Region (EMR) and South-East Asia Region (SEAR) was performed using Cytoscape, an open-source bioinformatics software platform used for depicting interaction networks^24^. We conducted further analysis on co-authorship networks generated using publications associated with EMR and SEAR grants, as these two regions have distinct tobacco use patterns, tobacco product types, and tobacco control activity. Publications associated with a grant that were not relevant to tobacco control or were not focused on the EMR or SEAR, were excluded.

## RESULTS

### Descriptive characteristics of international tobacco control grants

**Figure 1:**
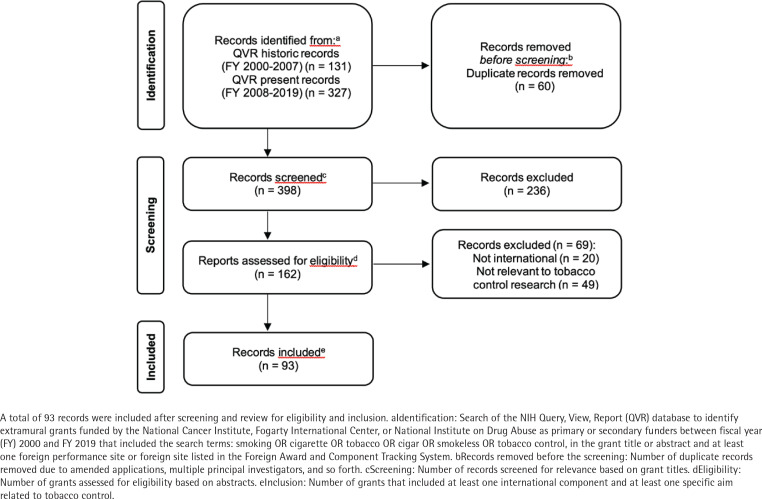
PRISMA flow diagram of the identification, screening, eligibility, and inclusion of grants for the portfolio analysis of National Institutes of Health (NIH)-funded global tobacco control research

We retrieved 458 extramural grants funded by NCI and/or FIC between FY 2000 and FY 2019. After removing 60 duplicate grants, we reviewed the titles of the remaining 398 grants for relevance to tobacco control research; 236 grants were deemed unrelated and were removed. As a final eligibility check, we reviewed the abstracts and specific aims of the remaining 162 grants for relevance to international tobacco control research. A total of 69 grants were excluded, as they did not have both an international component and at least one specific aim related to tobacco control. The remaining 93 grants were included in the portfolio analysis. Figure 1 shows the PRISMA^25^ flow diagram for grant inclusion in this portfolio analysis. Titles of all 93 grants are listed in Supplementary file Table S1. The descriptive characteristics of the 93 grants included in the international tobacco control research portfolio – funding agency, grant program, funding timeframe, geography, and income level – are shown in Table 1.

**Table 1:** Descriptive characteristics of international tobacco control grants (N=93)

*Variable*	*n*	*%*
**Funding agency/institute^a^**		
NCI	88	94.6
FIC	56	60.2
NIDA	52	55.9
NINR	11	11.8
NHLBI	5	5.4
NIMH	4	4.3
NIA	3	3.2
FDA	2	2.2
NIH OD	2	2.2
NIAAA	2	2.2
NINDS	2	2.2
NICHD	1	1.1
NIEHS	1	1.1
**Grant program^b^**		
Research project (R01)	66	71.0
Small grant (R03)	12	12.9
Exploratory (R21)	4	4.3
Academic enhancement (R15)	2	2.2
High-priority, short-term (R56)	1	1.1
Program (P01, P50)	4	4.3
Training (D43, K01, K07, R25)	4	4.3
**Fiscal year awarded^c^**		
2000–2004	25	26.9
2005–2009	28	30.1
2010–2014	20	21.5
2015–2019	20	21.5
**Contact PI institution location**		
United states	78	83.9
International	15	16.1

a Some grants were funded by more than one Institute; therefore, percentages exceed 100%.

b Details on National Institutes of Health (NIH) grant funding: https://grants.nih.gov/grants/funding/funding_program.htm.

c Indicates the first fiscal year in which the grant was awarded.

NCI: National Cancer Institute. FIC: Fogarty International Center. NIDA: National Institute on Drug Abuse. NINR: National Institute of Nursing Research. NHLBI: National Heart, Lung, and Blood Institute. NIMH: National Institute of Mental Health. NIA: National Institute on Aging. FDA: Food and Drug Administration. NIH OD: NIH Office of the Director. NIAAA: National Institute on Alcohol Abuse and Alcoholism. NINDS: National Institute of Neurological Disorders and Stroke. NICHD: National Institute on Child Health and Human Development. NIEHS: National Institute of Environmental Health Sciences.

*Funding agency*

Of the 93 grants in the portfolio, 94.6% received funding from NCI (n=88 grants), 60.2% from FIC (n=56 grants), 55.9% from NIDA (n=52 grants), and 11.8% from the National Institute of Nursing Research (n=11 grants). Note that these totals add up to greater than 93 as some grants were co-funded by more than one funding agency. Additional funding agencies are included in Table 1.

*Grant program*

Over 90% of grants in the portfolio were research grants, which included 70.9% large research projects (R01: 66); 12.3% small grants (R03: 12); 4.3% exploratory grants (R21: 4); smaller numbers of academic research enhancement awards (R15: 2); and high-priority, short-term awards (R56: 1). The portfolio also included 4.3% program grants (P01: 2, P50: 2) and 4.3% research training and career development grants (D43: 1, K01: 1, K07: 1, R25: 1).

*Fiscal year funding*

**Figure 2:**
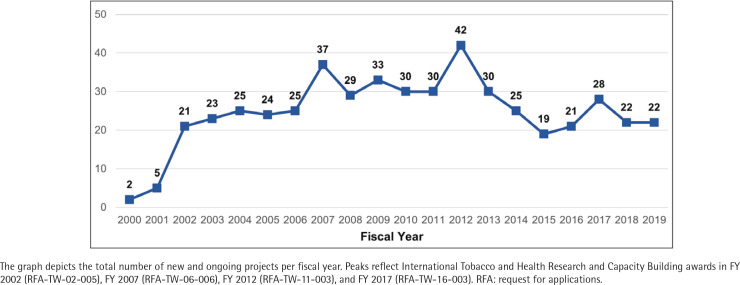
Number of active international tobacco control grants, by fiscal year

With the launch of the TOBAC program in 2002, the number of grants in the NIH portfolio increased substantially. Since 2003, there have been at least 19 active awards in the portfolio in any given fiscal year, with peaks coinciding with additional TOBAC awards in 2007, 2012, and 2017 (Figure 2). The TOBAC RFAs, supported by FIC, NCI, and NIDA, funded 44.1% of grants in the portfolio (n=41). The remaining grants were funded by tobacco-specific funding opportunity announcements (n=10; 10.8%), non-tobacco-specific funding opportunity announcements (n=39; 41.9%), or were investigator-initiated (n=3; 3.2%).

*PI institution and collaborator locations*

**Figure 3:**
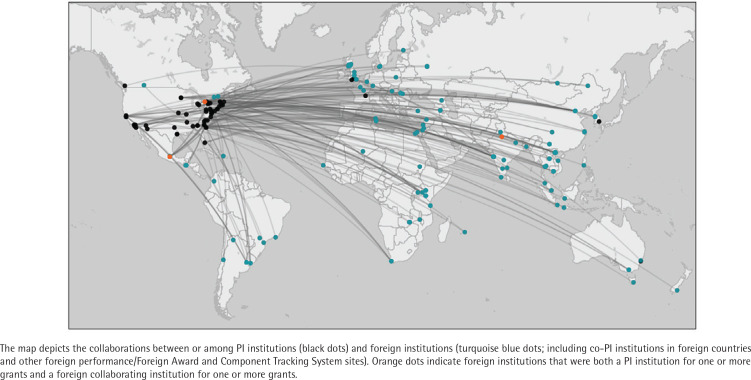
PI institutions and foreign collaborators

The 93 grants in the portfolio were awarded to 65 different PIs at 59 different institutions. Within the portfolio, 78 grants (83.9%) were awarded to PIs at US institutions, while 15 grants (16.1%) were awarded to PIs at foreign institutions in Canada (6 grants), United Kingdom (3 grants), India (2 grants), and Australia, France, Mexico, and South Korea (1 grant each). A total of 16 grants (17.2%) had multiple PIs; of these, 10 grants included co-PIs at foreign institutions (two grants with a co-PI in Vietnam, two grants with a co-PI in India, and one grant each with a co-PI in Argentina, Canada, China, Guatemala, Israel, and Mexico). Across all grants, there were 150 foreign collaborating sites in 51 countries (Figure 3).

### Focus and design of international tobacco control grants

*Country of focus*

The grants within the portfolio supported research focused on 53 foreign countries. Foreign countries appearing most frequently in the portfolio include India (n=16), China (n=15), and Canada (n=10). A complete list of foreign countries included as foci is included in Supplementary file Table S2.

*WHO region*

More than half (53.8%) of the grants supported research in Asia (SEAR and Western Pacific Region), 26.9% in the Americas Region, and 18.3% in the Europe Region. Significantly fewer grants supported research in the EMR (11.8%) and African Region (AFR) (10.8%).

**Table 2:** Focus and design of international tobacco control grants (N=93)

*Variable*	*n*	*%*
**WHO Region^a^**		
AFR	10	10.8
AMR	25	26.9
EMR	11	11.8
EUR	17	18.3
SEAR	21	22.6
WPR	29	31.2
Global	4	4.3
**Income level^b^**		
Low	17	18.3
Lower middle	45	48.4
Upper middle	33	35.5
High	22	23.7
**Tobacco product^c^**		
Cigarette	80	86.0
Hookah	8	8.6
Smokeless tobacco	5	5.4
Secondhand smoke	5	5.4
ENDS	3	3.2
Loose tobacco	1	1.1
IQOS	1	1.1
**Research focus^d^**		
Monitor (M)	45	48.4
Protect (P)	13	14.0
Offer (O)	30	32.3
Warn (W)	10	10.8
Enforce (E)	8	8.6
Raise (R)	9	9.7
Health effects	7	7.5
Tobacco industry	5	5.4
Tobacco products	3	3.2
Tobacco trade	2	2.2
Policy effects	2	2.2
Training	1	1.1
Other	2	2.2
**Research training component^e^**		
Research only	39	41.9
Research and training	54	58.1
**Study design^f^**		
Intervention	36	38.7
Survey	33	35.5
Qualitative	19	20.4
Epidemiological study	11	11.8
Laboratory study	11	11.8
Analysis of tobacco industry documents	8	8.6
Modeling	2	2.2
Secondary data analysis	2	2.2
Other	14	15.1

a Some grants coded for more than one country/WHO Region; therefore, the total percentage exceeds 100%.

b Some grants coded for more than one country/income level; therefore, the total percentage exceeds 100%. Four grants with a global focus were excluded from the income level table.

c Some grants coded for more than one tobacco product; therefore, the total percentage exceeds 100%.

d Some grants coded for more than one research focus; therefore, the total percentage exceeds 100%.

e All TOBAC grants included a training component, as required. For non-TOBAC grants, a training component was defined as a classroom program, a seminar, a workshop, a mentorship, or another activity designed to build skills in research, publication, or tobacco control, as described in the grant abstract.

f Some grants coded for more than one study design; therefore, the total percentage exceeds 100%.

AFR: African Region. AMR: Americas Region. EMR: Eastern Mediterranean Region. EUR: Europe Region. SEAR: South-East Asia Region. WPR: Western Pacific Region. ENDS: Electronic Nicotine Delivery System.

*Income level*

More than 80% of grants supported research in upper middle-income countries (35.5%) or lower middle-income countries (48.4%). Almost one-quarter of grants (23.7%) supported research in HICs, and only 18.3% supported research in low-income countries (LICs). Over time, the number of grants in LICs decreased, in part because economic growth boosted several countries (e.g. India, Indonesia) from the low-income to lower middle-income bracket.

*Tobacco product*

The majority of grants (86.0%) focused on cigarettes, while 23.7% of the grants (22 unique grants) included a focus on any other tobacco product, such as waterpipe, smokeless tobacco, secondhand smoke, e-cigarettes, loose tobacco, or IQOS. Similarly, in every region, cigarettes were by far the most frequently studied product, except for the EMR, where more than 70% of studies included a focus on waterpipe tobacco smoking (Regional data are available in Supplementary file Table S3).

*Research focus*

Most grants (89.2%) focused on at least one of the MPOWER tobacco control policy measures in the WHO MPOWER package. Almost half of the portfolio grants (48.4%) included a focus on monitoring tobacco use and prevention policies (*Monitor*). About one-third (32.3%) included a focus on tobacco cessation (*Offer*). The remaining MPOWER measures (*Protect, Warn, Enforce, Raise*) were addressed in only 8–14% of grants. This pattern was also observed at the regional level. *Monitor* was the most frequently studied MPOWER measure across WHO regions, except for AFR, where an equal number of grants addressed both *Monitor* and tax issues (*Raise*). (Regional data are available in Supplementary file Table S4). In addition, 7.5% of grants focused on health effects, 5.4% on tobacco industry interference, 2.2% on tobacco trade, and 2.2% on policy effects.

*Training*

Approximately 4% of portfolio grants were funded through NIH Institutional Training and Education Grant mechanisms (D43, K01, K07, R25). However, many grants (58.1%) included a research training component, for example, a classroom program, a seminar, a workshop, a mentorship, or another activity designed to build skills in the areas of research, publication, or tobacco control.

*Study design*

The most common study designs in the portfolio were interventions (38.7% of grants) and surveys (35.5% of grants). Qualitative, epidemiological, and laboratory study designs were also common.

*Populations of special interest*

Within the grant portfolio, 28 grants (30.1%) included a focus on tobacco use, exposure, or cessation among one or more special or vulnerable populations. These grants focused on youths/young adults (18 grants), SES (5 grants), maternal and child health (5 grants), health professionals (2 grants), and communicable diseases (1 grant).

### Publications

We identified 85 grantees who reported at least one publication as of 17 July 2020. Seven grants had more than 100 publications associated with them, with one grant reporting 264 associated publications. Eight grants, six of which were awarded in the last four years, reported no publications at the time of data extraction. In total, grants were associated with 2386 publications (mean per grant=28, median=7, range: 0–264), with duplicates possible across grants. After removing the duplicates, there were 1873 publications across 390 journals. The most highly represented journals were: Tobacco Control (358 articles), Nicotine and Tobacco Research (153 articles), Addiction (64), American Journal of Public Health (58), and International Journal of Environmental Research and Public Health (45). Between 2000 and 2020, an average of nearly 94 articles were published per year. Grants related to the International Tobacco Control Policy Evaluation Project – an ongoing cohort study active in 31 countries that began in 2002 and has received funding from multiple sources, including NIH – accounted for 367 unique publications.

### Co-authorship network analysis

**Figure 4:**
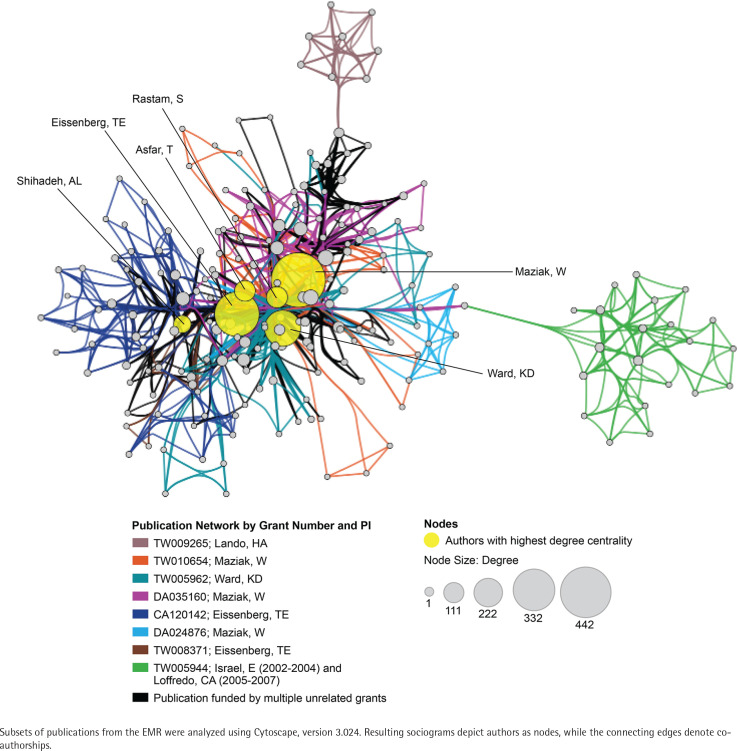
Co-authorship network analysis for the WHO Eastern Mediterranean Region (EMR)

**Figure 5:**
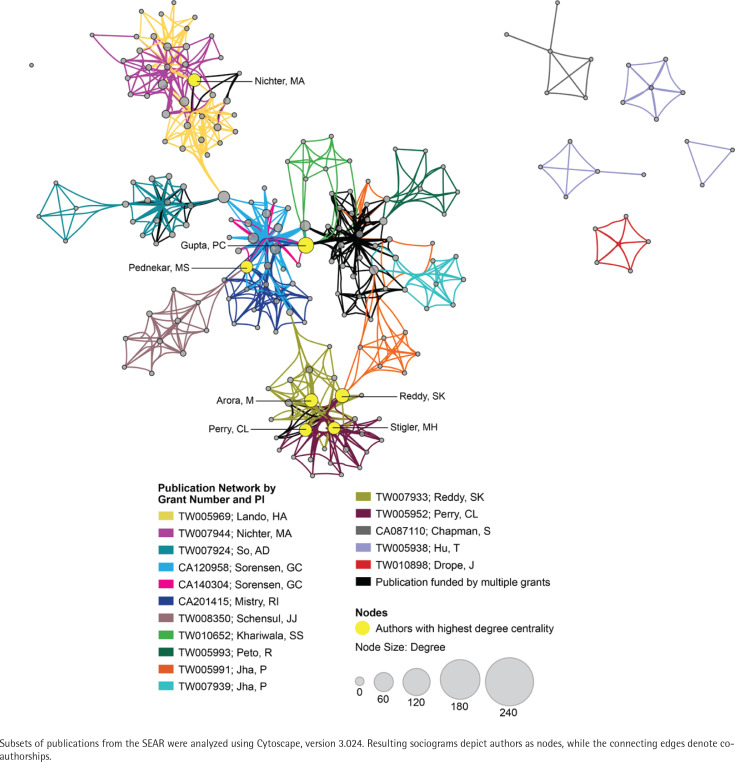
Co-authorship network analysis for the WHO South-East Asia Region (SEAR)

The EMR 2020 co-authorship network (Figure 4) consists of 141 publications from 9 grants. The network includes 202 nodes (authors), 2123 edges (co-authorships), 1 component (distinct group of connected authors), and 0 isolated nodes; the network diameter is 6, density is 0.054, and centralization is 0.509 (Table 3). In contrast, the SEAR 2020 co-authorship network (Figure 5) consists of 85 publications from 16 grants. The network includes 202 nodes, 1798 edges, seven components, and one isolated node; the network diameter is 5, density is 0.055, and centralization is 0.266 (Table 3).

**Table 3:** Co-authorship network analysis: network characteristics, 2007 vs 2020

*Network characteristics*	*EMR 2007*	*EMR 2020*	*SEAR 2007*	*SEAR 2020*
Grants	2	9	6	16
Publications	26	141	16	85
Nodes (authors)	38	202	55	202
Edges (co-authorships)	405	2123	237	1798
Connected components	2	1	5	7
Nodes in largest connected component	32	202	23	176
Isolated nodes	0	0	0	1
Diameter^a^	3	6	4	5
Density^b^	0.245	0.054	0.139	0.055
Centralization^c^	0.541	0.509	0.163	0.266

a Diameter: shortest path length (distance) between the two most distant nodes in the network.

b Density: ratio of the number of edges and the number of possible edges, range 0–1.

c Centralization: extent to which the connections of a given network are concentrated on a single node or group of nodes, range 0–1.

EMR: Eastern Mediterranean Region. SEAR: South-East Asia Region.

## DISCUSSION

This article, covering a 20-year period, provides the first systematic portfolio analysis of global tobacco control research funded at NCI. Out of 93 grants included in the analysis, the majority were NCI-funded R01 Research Project Grants (71%), and almost half of all grants (44%) were funded under the FIC-led TOBAC program. Although research activity occurred across 53 foreign (non-US) countries, 97.8% (91) of the grants were awarded to institutions in HICs (primarily the US, Canada, and the UK), with only three grants awarded directly to LMIC institutions in two countries (India and Mexico). More than half (53.8%) of research grants included activity in Asia, while the EMR and AFR were the least represented in the grant portfolio.

Some areas of research focus received greater attention within the portfolio. Almost half the grants (48.4%) included a focus on *Monitoring* tobacco use and prevention policies. *Monitoring* is an essential component of both WHO FCTC and MPOWER, as having adequate data on prevalence is needed to understand the scope of tobacco use, determine the resources needed to address tobacco use and evaluate the impact of policies and interventions at the population level. A focus on monitoring may be especially appropriate early on in a research initiative to characterize the tobacco epidemic within a given context. However, the critical first step of monitoring can be enhanced by being linked to additional research and action around the development and implementation of interventions. Cessation (*Offer*) was another strong focus observed in the research portfolio. Context-specific research to study the implementation of tobacco cessation interventions and strategies is a high priority, particularly for LMICs, where a strong infrastructure for the delivery of cessation services may be lacking^26^. Although many countries report offering cessation services, these services do not necessarily meet WHO guidelines for WHO FCTC implementation at the national level^27^. There is a need for continued research to develop or adapt and implement novel, low-cost cessation interventions in LMICs.

Going forward, the focus of research could shift toward the study of the implementation of tobacco control interventions in LMIC settings and ways to scale up effective interventions.

The majority of grants (86.0%) focused on cigarettes. Although important ongoing research programs exist around non-cigarette tobacco products, such as work on waterpipe tobacco smoking in the EMR and smokeless tobacco use in India, these projects make up a relatively small portion of the overall portfolio. Given the high prevalence of the use of non-cigarette tobacco products in some populations, such as smokeless tobacco use among women in India^28^, this disparity could be addressed in future research programs. Moreover, research on emerging global trends may have relevance for the United States. For example, waterpipe tobacco research conducted in the Middle East provided important data to inform the US response to rising waterpipe tobacco smoking among US youths^29-31^. As products, including ENDS and heated tobacco products, emerge on the global market under different regulatory and market conditions, there are critical opportunities to learn from experiences across diverse settings in real-time.

Our analysis also highlighted regional gaps in tobacco control research, particularly among LICs and in the EMR and AFR, where the prevalence of tobacco use has decreased, but the overall number of tobacco smokers is projected to increase due to population growth^3^. Building research capacity and activity in these regions could be a focus for future work. There have been some recent efforts to increase and develop priorities for tobacco control research in Africa. The Center for Tobacco Control in Africa, through an ongoing consultation process, identified eight central research themes: 1) patterns of tobacco use, 2) effects of use (including impacts on sustainable development goals), 3) populations at risk, 4) policy and implementation, 5) the sociocultural context of tobacco use, 6) the tobacco industry, 7) tobacco production and alternative livelihoods, and 8) the economics of tobacco and tobacco control.^32^ Additionally, few grants included a research focus on tobacco industry interference or on tobacco growing and economics, which are issues particularly important for African countries, where tobacco growing is a substantial contributor to their national economies^2,32^.

In terms of publications, this NIH-funded portfolio of international tobacco grants was remarkably productive, with an average of 94 publications per year between 2000 and 2020. One grant reported 264 publications, and seven grants reported more than 100 publications. Only eight grants, six of which were awarded in the past four years, reported no publications at the time of data extraction. There can be multiple reasons for the differences in the numbers of publications across the grant portfolio, but it is important to note that more recent grants will show fewer publications, as publications may continue to appear for several years after the grant funding period has ended. The mean RCR for the portfolio of international tobacco control grants as of August 2022 was 1.92, which suggests that the publications within this grant portfolio had an above-average influence relative to other NIH-funded articles^23,33^.

Given the high prevalence of non-cigarette tobacco (waterpipe tobacco smoking) and smokeless tobacco use in the EMR and SEAR, we looked at the change in co-authorship networks over time. Over the 20-year portfolio period, both the EMR and SEAR networks expanded (in terms of numbers of grants, publications, and co-authorships) and matured (in terms of diameter, density, and centrality). There was a 2.7- to 4.5-fold increase in the number of grants in the SEAR and EMR, respectively, which is encouraging given the low representation of EMR countries in the overall portfolio. EMR grantees were slightly more prolific, generating 15.7 publications per grant compared with 5.3 publications per SEAR grant; however, the SEAR grantees had slightly more co-authorships per publication (21 vs 15). In general, the EMR co-authorship network is more centralized than the SEAR co-authorship network (0.51 vs 0.27), perhaps reflecting a greater research focus on waterpipe tobacco smoking in EMR compared with smokeless tobacco use in SEAR.

The finding that only 3 of 93 grants were awarded directly to LMIC investigators raises important questions of equity. Over the past two years, there has been increased attention to inequities in funding and collaborative structures in global health, along with calls for ‘decolonizing’ global health^34^. The TOBAC program used a model pairing US institutions with LMIC institutions, where the US institution served as the primary grantee institution, providing mentorship and capacity-building assistance, and the research activity and funds (>50% for awards made in 2002, 2007, and 2012) were focused on the LMIC institution^16^. Research support and capacity-building needs in LMICs have evolved over the past two decades with the impact of the FCTC and the support of global funders. Today, a growing number of LMIC institutions have developed active research programs in tobacco control and other areas of cancer control and offer their own regional training and capacity-building programs^35-38^. The NCI Center for Global Health holds equity as a core value, as outlined in its current Strategic Plan, and has emphasized the importance of equitable leadership plans in recent funding announcements^39^.

### Limitations

This analysis focused on extramural grants that were funded from FY 2000 to FY 2019 and may not represent all global tobacco research funded by NIH. The characterization of the grant research focus and design was based solely on the application title and abstract. Data for grant-associated publications were based on investigators’ self-reports.

## CONCLUSIONS

Although tremendous progress has been made over the past two decades in advancing tobacco control policies and programs around the world, the evolving nature of the global tobacco epidemic highlights the critical need for further research to encourage the adaptation, adoption, integration, and scale-up of evidence-based interventions across diverse LMIC settings, with a goal of full implementation of the WHO FCTC and related tobacco control measures.

## Supplementary Material



## Data Availability

The data used in this study were obtained from the QVR system, which is an internal NIH database. Publicly available data regarding the NIH-funded grants in this analysis are available from NIH RePORTER: https://reporter.nih.gov/.
