# Supplementary material for: Portfolio analysis of global tobacco control research funding at the National Cancer Institute, 2000–2019
Source: Tob Prev Cessat. 2024 Mar 4;10:10.18332/tpc/184041. doi: 10.18332/tpc/184041 (PMC10910547; doi:10.18332/tpc/184041)
Supplement: Supplementary file 1 [file TPC-10-13-s1.pdf]

Supplementary Table 1: Global Tobacco Control Grants included in Portfolio Analysis

Full Grant Number	Project Title	NIH Reporter Link
D43TW009333-01	Cancer and Tobacco Control Training and Research Across the Lifespan In Kenya	https://reporter.nih.gov/project-details/8337683
K01TW009654-01A1	Family Smoking Cessation in Romania Using Pregnancy as a Window of Opportunity	https://reporter.nih.gov/project-details/8816582
K07CA093605-01A2	Learning from Others: Smoking Cessation in Urban China	https://reporter.nih.gov/project-details/6596552
P01CA138389-01	Effectiveness of Tobacco Control Policies in High vs. Low Income Countries	https://reporter.nih.gov/project-details/7632346
P01CA200512-01	Evaluating How Tobacco Control Policies are Shaping the Nicotine Delivery Market	https://reporter.nih.gov/project-details/8998639
P50CA084735-06	Genes, Environment & Tobacco Use Across Cultures	https://reporter.nih.gov/project-details/6847948
P50CA111236-01	Building the Evidence Base for Tobacco Control Policies	https://reporter.nih.gov/project-details/6847733
R01CA087110-01A1	Analysis of Tobacco Industry Documents--Asia/Australia	https://reporter.nih.gov/project-details/6323887
R01CA087472-01	Analysis of Tobacco Industry Documents	https://reporter.nih.gov/project-details/6174481
R01CA090955-01A1	Effects of Graphic Warning Labels on Adolescent Smoking	https://reporter.nih.gov/project-details/6435739
R01CA091021-01	Globalization, the Tobacco Industry and Policy Influence; Tobacco Companies, Public Policy and Global Health	https://reporter.nih.gov/project-details/6324059
R01CA092039-01A2	Genetics of Tobacco and Alcohol Related Cancers	https://reporter.nih.gov/project-details/6685047
R01CA100362	International Tobacco Policy Survey/International Tobacco Control Policy Survey	https://reporter.nih.gov/project-details/8546965
R01CA117108-01A1	Evaluating Low Ignition Propensity Cigarette Legislation	https://reporter.nih.gov/project-details/7144399
R01CA120138-06A1	Investigating Institutional Influences on Tobacco Control	https://reporter.nih.gov/project-details/8579482
R01CA120142-01A2	Waterpipe Tobacco Smoke: Toxicant Exposure and Effects	https://reporter.nih.gov/project-details/7370210
R01CA120958-01A1	Promoting Tobacco Control Among Teachers in India	https://reporter.nih.gov/project-details/7209156
R01CA125116-01A1	Tobacco Control Policy Evaluation in China and South Korea: The ITC Asia Project	https://reporter.nih.gov/project-details/7321408
R01CA128638-01A1	Knowledge Integration in Quitlines: Networks that Improve Cessation	https://reporter.nih.gov/project-details/7431848
R01CA129534-01A2	Biomarkers of NNK in Prediction of Lung Cancer Development in Smokers	https://reporter.nih.gov/project-details/7729808
R01CA132950-01A2	Community-Partnered Tobacco Control in Underserved Dominican Republic Communities	https://reporter.nih.gov/project-details/7730018

R01CA140304-01A1	Mumbai Worksite Tobacco Control Study	https://reporter.nih.gov/project-details/7886013
R01CA157577-01A1	The Global Diffusion of Tobacco Control	https://reporter.nih.gov/project-details/8263899
R01CA160695-01	Tobacco Companies, Global Governance and Public Health	https://reporter.nih.gov/project-details/8163761
R01CA167067-01A1	Building Evidence for Effective and Sustainable Cigarette Warning Label Policy	https://reporter.nih.gov/project-details/8399830
R01CA175329-01A1	Implementing Tobacco Use Treatment Guidelines in Community Health Centers In Viet	https://reporter.nih.gov/project-details/8631772
R01CA200691-01A1	Disseminating an Evidence-Based Tobacco Control Intervention for School Teachers In India	https://reporter.nih.gov/project-details/9238131
R01CA201415-01A1	Longitudinal Study of Adolescent Tobacco Use and Tobacco Control Policy in India	https://reporter.nih.gov/project-details/9175358
R01CA215466-01A1	Evaluation Of Cigarette Package Inserts for Enhanced Communication With Smokers	https://reporter.nih.gov/project-details/9668457
R01CA225419-01A1	Optimizing Smoking Cessation Interventions for PLWH in Nairobi, Kenya	https://reporter.nih.gov/project-details/9616972
R01CA239178-01A1	Assessing IQOS Marketing Influences and Consumer Behavior in Israel: Implications for the US	https://reporter.nih.gov/project-details/9872508
R01DA012854-01	The Genetics of Vulnerability to Nicotine Addictions	https://reporter.nih.gov/project-details/6024273
R01DA024875-01	Network for Tobacco Control among Women in Parana, Brazil	https://reporter.nih.gov/project-details/7293093
R01DA024876-02	Responding to the Changing Tobacco Epidemic in the Eastern Mediterranean Region	https://reporter.nih.gov/project-details/7500669
R01DA024877-07	Tobacco Control Research and Training in South America	https://reporter.nih.gov/project-details/7500672
R01DA035157-01	Preventing Tobacco Use Among Adolescents In Uruguay: Project Activate	https://reporter.nih.gov/project-details/8332442
R01DA035158-01	Building Research and Capacity on the Economic Policy-Tobacco Control Nexus in Africa	https://reporter.nih.gov/project-details/8333680
R01DA035160-06	Advancing Addiction Research and Capacity to Guide Tobacco Control in the Eastern	https://reporter.nih.gov/project-details/8332504
R01DA035384-01	Smoking Cessation Contracts with Social and Monetary Incentives	https://reporter.nih.gov/project-details/8483234
R01HL073699-01	Epidemiology & Intervention Research for Tobacco Control	https://reporter.nih.gov/project-details/6541109
R01TW005931-01	South Africa Adolescent Smoking: A Longitudinal Study	https://reporter.nih.gov/project-details/6744996
R01TW005935-01	Tobacco Use Among Argentinian Youth: A Cohort Study	https://reporter.nih.gov/project-details/6540999

R01TW005938-01	Tobacco Control Policy Analysis & Intervention Evaluation in China and Indonesia	https://reporter.nih.gov/project-details/6535934
R01TW005944-01	Egypt Smoking Prevention Research Initiative	https://reporter.nih.gov/project-details/6540956
R01TW005945-01	Technology Assisted Dominican Republic Tobacco Control	https://reporter.nih.gov/project-details/6540970
R01TW005952-01	Mobilizing Youth for Action Against Tobacco in India	https://reporter.nih.gov/project-details/6743572
R01TW005955-01	PSU-Western & Southern African Tobacco Research Project	https://reporter.nih.gov/project-details/6802923
R01TW005962-01	Establishment of the Syrian Center for Tobacco Studies	https://reporter.nih.gov/project-details/6540929
R01TW005964-01	Asian Leadership Training for Tobacco Control Research	https://reporter.nih.gov/project-details/6541096
R01TW005964-06	Building GIS Capacity into Tobacco Control Research Programs of East Asia	https://reporter.nih.gov/project-details/9372582
R01TW005969-01	Cessation Research and Training in India and Indonesia	https://reporter.nih.gov/project-details/6541076
R01TW005977-01	Tobacco Control in S.Africa: Prevention and Capacity Building	https://reporter.nih.gov/project-details/6540994
R01TW005991-01	Strengthening Monitoring of Indian Tobacco Mortality	https://reporter.nih.gov/project-details/6546488
R01TW005993-01	Monitoring Tobacco Mortality in 2M Adults in 4 Countries	https://reporter.nih.gov/project-details/6547123
R01TW007918-01	SMS Turkey: Harnessing the Power of TXT Messaging to Promote Smoking Cessation	https://reporter.nih.gov/project-details/7292953
R01TW007924-01	The Political Economy of Tobacco Control in Southeast Asia	https://reporter.nih.gov/project-details/7293331
R01TW007927-01	Increasing Capacity for Tobacco Research in Hungary	https://reporter.nih.gov/project-details/7293165
R01TW007933-01	Advancing Cessation of Tobacco In Vulnerable Indian Tobacco consuming Youth	https://reporter.nih.gov/project-details/7292970
R01TW007939-01	Measuring Tobacco Mortality within the Million Death Study in India	https://reporter.nih.gov/project-details/7293028
R01TW007944-01	Building Capacity of Tobacco Cessation in India & Indonesia	https://reporter.nih.gov/project-details/7293089
R01TW007949-06	Epidemiology & Intervention Research for Tobacco Control in China	https://reporter.nih.gov/project-details/7293019
R01TW009272-01	Tobacco Control Network among Women in Parana, Brazil - II	https://reporter.nih.gov/project-details/8332634
R01TW009274-01	Cinema Smoking and Youth Smoking in Latin America	https://reporter.nih.gov/project-details/8333037
R01TW009280-01	Building Capacity for Tobacco Research in Romania	https://reporter.nih.gov/project-details/8333525
R01TW009288-01	From Production to Retailing: Policy-Oriented Research on Tobacco Economy In Argentina	https://reporter.nih.gov/project-details/8333929
R01TW009295-01	Tobacco Control Policy Analysis & Intervention Evaluation in China and Tanzania	https://reporter.nih.gov/project-details/8333920

R01TW010647-01	mHealth Messaging to Motivate Quitline Use and Quitting (M2Q2): RCT in Rural Vietnam	https://reporter.nih.gov/project-details/9371591
R01TW010651-01	Analytical Capacity Building for the Study of Tobacco Carcinogen Exposures in India	https://reporter.nih.gov/project-details/9371941
R01TW010652-01	Electronic Cigarettes in Latin America: Evaluation of Impacts and Policy Options	https://reporter.nih.gov/project-details/9371943
R01TW010654-01	Translating Evidence and Building Capacity to Support Waterpipe Control In The Eastern Mediterranean	https://reporter.nih.gov/project-details/9371991
R01TW010664-01	Smoke-free Air Coalitions in Georgia and Armenia: A Community Randomized Trial	https://reporter.nih.gov/project-details/9372431
R01TW010666-01	Cultural Adaptation and Evaluation of Health Interventions for Smoking Cessation in China and Vietnam	https://reporter.nih.gov/project-details/9372474
R01TW010898-06	The Political Economy of Tobacco Farming in Low-and Middle-Income Countries	https://reporter.nih.gov/project-details/9372362
R03CA094771-01	A Cohort Study of Active and Passive Smoking in Korea	https://reporter.nih.gov/project-details/6448550
R03CA130728-01	Susceptibility to Smoking Initiation Among Rural and Urban Young Chinese Women	https://reporter.nih.gov/project-details/7336742
R03CA219434-01A1	Consequences of HELQ Alterations in Alcohol And Tobacco-Related Cancers	https://reporter.nih.gov/project-details/9595753
R03TW005692-01	Clusters of Drug Involvement in Chile	https://reporter.nih.gov/project-details/6402324
R03TW007164-01A1	Biobehavioral Model of Smoking by Russians with Cancer	https://reporter.nih.gov/project-details/6988428
R03TW007233-01A1	Establishment of the Syrian Center for Cancer	https://reporter.nih.gov/project-details/7125865
R03TW007345-01	Design and Validation of a Tobacco Survey	https://reporter.nih.gov/project-details/6988987
R03TW008350-01	Smokeless Tobacco Use and Reproductive Health Among Married Women in a Low-income	https://reporter.nih.gov/project-details/7694646
R03TW008361-01	Susceptibility to Smoking Initiation Among Young Rural-Urban Migrants in China	https://reporter.nih.gov/project-details/7694837
R03TW008371-01	Waterpipe Tobacco Smoking: Trends, Toxicants, and Capacity Building In Jordan	https://reporter.nih.gov/project-details/7697276
R03TW008723-01	Optimizing Treatment for Brazilian Smokers through Community-Based Primary Care	https://reporter.nih.gov/project-details/7943750
R03TW008962-01	Assessment Of Causality Between Exposure To Tobacco Use In Bollywood Movies	https://reporter.nih.gov/project-details/7944369

R15CA133152-01	Moralization, Risk Perceptions, and Smoking Cessation in the U.S. and Denmark	https://reporter.nih.gov/project-details/7447145
R15CA194937-01A1	The Effects of Stigmatizing US and Danish Smokers	https://reporter.nih.gov/project-details/9021467
R21CA225852-01	Feasibility and Acceptability of a Text Messaging Intervention to Increase Smoking Cessation in Vietnam	https://reporter.nih.gov/project-details/9334431
R21DA047358-01	Evaluation of Canada's Menthol Ban	https://reporter.nih.gov/project-details/9646550
R21TW010637-01	Network for Tobacco Control Among Women in Antioquia, Colombia	https://reporter.nih.gov/project-details/9231233
R21TW010896-01	Mobile Health Intervention for Family Smoking Cessation In Romania	https://reporter.nih.gov/project-details/9340349
R25TW008112-01	Framework Program for Global Health in the Americas	https://reporter.nih.gov/project-details/7499899
R56TW009265-01	Capacity Building for Tobacco Control in Tunisia, North Africa & Middle East	https://reporter.nih.gov/project-details/8331840

Supplementary Table 2: Grants by Country of Focus

Country	No. Grants	% Grants
India	16	17.2%
China	15	16.1%
Canada	10	10.8%
Australia	8	8.6%
Mexico	8	8.6%
Indonesia	6	6.5%
Kenya	5	5.4%
South Korea	5	5.4%
Thailand	5	5.4%
Vietnam	5	5.4%
Argentina	4	4.3%
Brazil	4	4.3%
Laos	4	4.3%
Lebanon	4	4.3%
Malaysia	4	4.3%
Syria	4	4.3%
United Kingdom	4	4.3%
Cambodia	3	4.3%
Egypt	3	3.2%
France	3	3.2%
Romania	3	3.2%
South Africa	3	3.2%
Uruguay	3	3.2%
Zambia	3	3.2%
Denmark	2	3.2%
Dominican Republic	2	2.2%
Hungary	2	2.2%
Jordan	2	2.2%
Malawi	2	2.2%
New Zealand	2	2.2%
Russia	2	2.2%
Tanzania	2	2.2%
Tunisia	2	2.2%
Armenia	1	2.2%
Bangladesh	1	1.1%
Benin	1	1.1%
Cameroon	1	1.1%
Chile	1	1.1%
Colombia	1	1.1%
Finland	1	1.1%

Georgia	1	1.1%
Germany	1	1.1%
Guatemala	1	1.1%
Israel	1	1.1%
Mauritius	1	1.1%
Mongolia	1	1.1%
Netherlands	1	1.1%
Philippines	1	1.1%
Poland	1	1.1%
Senegal	1	1.1%
Singapore	1	1.1%
Turkey	1	1.1%
Uganda	1	1.1%

Supplementary Table 3: Grants by WHO Region and Tobacco Product Focus

**WHO Region**	**Cigarettes**		**ENDS**		**Hookah**		**IQOS**		**Loose Tobacco**		**Secondhand Smoke**		**Smokeless Tobacco**	
	No.	%	No.	%	No.	%	No.	%	No.	%	No.	%	No.	%
AFR	10	10.8%	-	-	-	-	-	-	-	-	-	-	-	-
AMR	23	24.7%	2	2.2%	-	-	-	-	-	-	2	2.2%	-	-
EMR	5	5.4%	-	-	8	8.6%	-	-	-	-	-	-	-	-
EUR	14	15.1%	1	1.1%	-	-	1	1.1%	-	-	1	1.1%		
SEAR	19	20.4%	1	1.1%	-	-	-	-	-	-	-	-	5	5.4%
WPR	27	29.0%	1	1.1%	-	-	-	-	1	1.1%	1	1.1%	-	-
Global focus	4	4.3%	-	-	-	-	-	-	-	-	1	1.1%	-	-

AFR=African Region. AMR=Region of the Americas. European Region=European Region. EMR=Eastern Mediterranean Region. WPR=Western Pacific Region.

Supplementary Table 4: Grants by WHO Region and Research Focus

WHO Region	MPOWER Research Focus													
	Monitor		Protect		Offer		Warn		Enforce		Raise			
	No.	%	No.	%	No.	%	No.	%	No.	%	No.	%		
AFR	3	3.6%	1	1.2%	2	2.4%	2	2.4%	1	1.2%	3	3.6%		
AMR	12	14.5%	7	8.4%	7	8.4%	6	7.2%	3	3.6%	2	2.4%		
EMR	8	9.6%	1	1.2%	4	4.8%	1	1.2%	-	-	-	-		
EUR	10	12.0%	4	4.8%	4	4.8%	2	2.4%	2	2.4%	1	1.2%		
SEAR	10	12.0%	2	2.4%	8	9.6%	2	2.4%	1	1.2%	2	2.4%		
WPR	16	19.3%	6	7.2%	5	6.0%	6	7.2%	4	4.8%	5	6.0%		
Global focus	1	1.2%	-	-	-	-	-	-	-	-	1	1.2%		
	**Additional Research Focus Categories**													
	**Health Effects**		**Tobacco Industry**		**Tobacco Products**		**Tobacco Trade**		**Policy Effects**		**Training**		**Other**	
	No.	%	No.	%	No.	%	No.	%	No.	%	No.	%	No.	%
AFR	-	-	1	4.8%	-	-	1	4.8%	-	-	-	-	-	-
AMR	-	-	1	4.8%	2	9.5%	-	-	2	9.5%	1	4.8%	-	-
EMR	1	4.8%	-	-	1	4.8%	-	-	1	4.8%	-	-	-	-
EUR	-	-	-	-	-	-	-	-	2	9.5%	-	-	-	-
SEAR	3	14.3%	1	4.8%	-	-	1	4.8%	1	4.8%	-	-	-	-
WPR	3	14.3%	1	4.8%	-	-	-	-	2	9.5%	-	-	1	4.8%
Global focus	-	-	2	9.5%	-	-	1	4.8%	-	-	-	-	1	4.8%

AFR=African Region. AMR=Region of the Americas. European Region=European Region. EMR=Eastern Mediterranean Region. WPR=Western Pacific Region.

© 2024 Ricciardone M. D. et al.
